# Supplementary material for: Nitric Oxide Overproduction by cue1 Mutants Differs on Developmental Stages and Growth Conditions
Source: Plants (Basel). 2020 Nov 4;9(11):1484. doi: 10.3390/plants9111484 (PMC7692804; doi:10.3390/plants9111484)
Supplement: Supplementary file 1 [file plants-09-01484-s001.pdf]

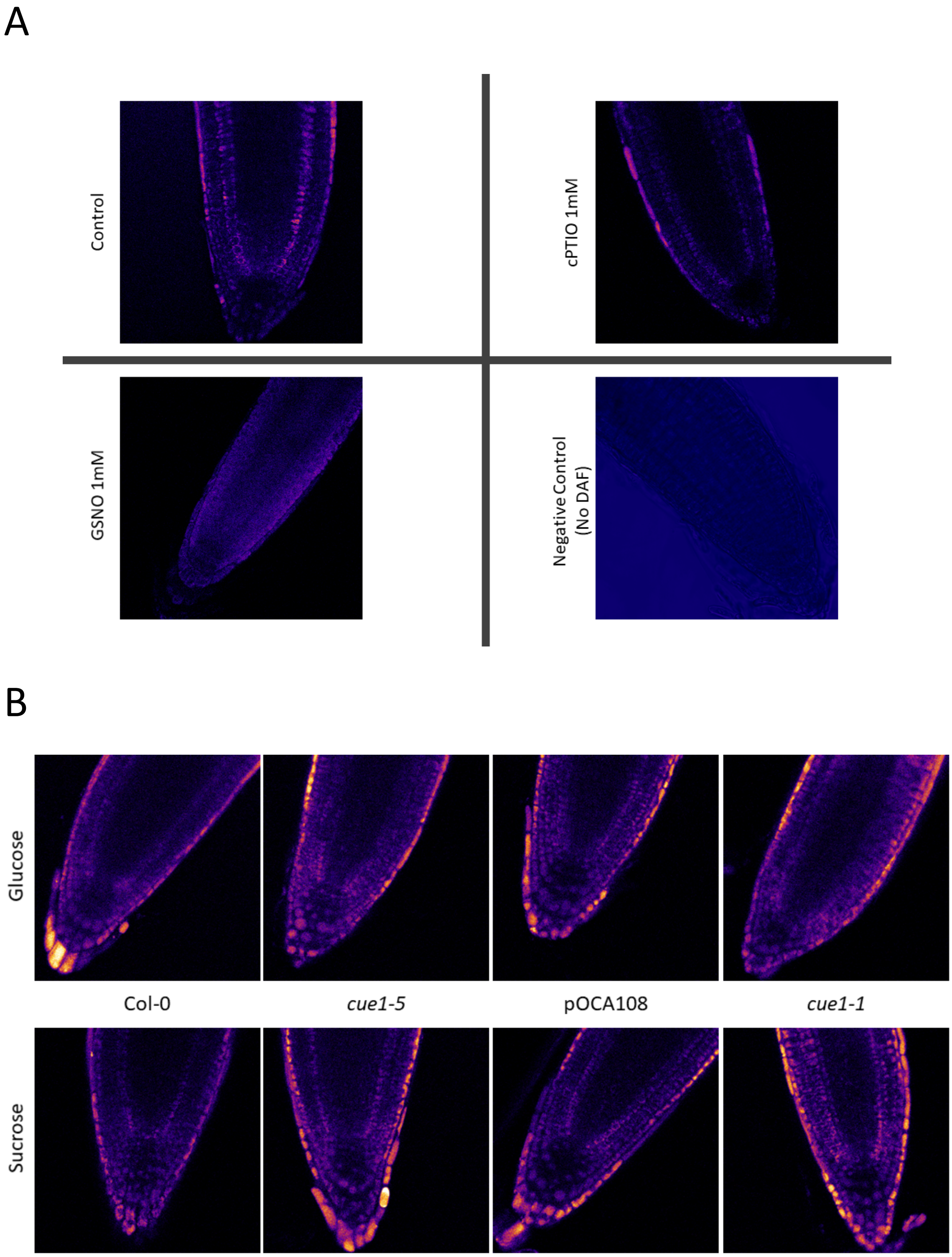

Figure S1. NO content by *in vivo* imaging. (A) Detection of NO by confocal microscopy using root tips of 7-day-old Col-0 seedlings stained with DAF-FM DA incubation after treatment with NO scavenger (cPTIO) and donor (GSNO). cPTIO is able to scavenge DAF and GSNO increases local maxima. (B) DAF fluorescence by confocal microscopy using root tips of 7-day-old seedlings from Col-0, pOCA108 and *cue1* alleles grown on MS-Root medium supplemented with either 2%(w/v) glucose or 0.75%(w/v) sucrose. FIRE LUT was used as a fluorescence heatmap.

Table S1. Sugar and NO affect root meristem size. Meristem size parameters of 7-day-old seedlings grown on MS-Root medium supplemented with either 2% glucose or 0.75% sucrose. Values represent the mean ± CI (N = 104). All units were in µm and 10 roots were analyzed per genotype and carbon source.

	Glucose				Sucrose			
	Col-0	*cue1-5*	pOCA	*cue1-1*	Col-0	*cue1-5*	pOCA	*cue1-1*
Root Length (mm)	17.26 ± 0.41	8.61 ± 0.94	22.03 ± 0.74	10.06 ± 0.67	17.75 ± 0.51	12.00 ± 0.58	18.52 ± 0.56	10.97 ± 0.59
Root Elongation Rate (mm/d)	3.81 ± 0.28	2.00 ± 0.83	4.95 ± 0.47	2.30 ± 0.32	3.75 ± 0.88	2.76 ± 0.57	4.10 ± 0.62	2.48 ± 0.52
Meristem Size (µm)	192.92 ±23.05	153.36 ±13.01	185.20 ± 14.09	157.58 ± 17.85	199.27 ± 18.43	163.83 ± 19.34	206.57 ± 21.08	203.21 ± 29.20
Meristematic Cell Number (cells)	24.8 ± 2.8	17.4 ± 1.7	23.4 ± 1.6	16.4 ± 2.3	26.1 ± 1.2	20.7 ± 2.7	30.0 ± 2.6	25.4 ± 1.2
Meristematic Cell Length (µm)	8.16 ± 0.38	8.87 ± 0.73	7.95 ± 0.47	9.74 ± 0.77	7.72 ± 0.36	7.98 ± 0.37	6.89 ± 0.42	7.98 ± 0.97
Average Cell Length Cells 1-10	7.93 ± 0.54	8.63 ± 0.76	7.77 ± 0.52	9.37 ± 0.60	7.01 ± 0.29	7.44 ± 0.42	7.01 ± 0.56	7.5 ± 0.60
Average Cell Length Cells 11-20	7.83 ± 0.45	11.94 ± 2.39	7.76 ± 0.63	13.94 ± 3.12	6.95 ± 0.36	9.11 ± 1.45	6.39 ± 1.18	7.46 ± 1.15
Average Cell Length Cells 21-30	15.03 ± 2.59	33.28 ± 9.41	14.55 ± 2.05	29.51 ± 6.33	14.87 ± 1.57	20.40 ± 3.80	13.16 ± 8.78	14.35 ± 3.70

Table S2. Two-way ANOVA statistical parameters and simple main effects results.

ANOVA Table (type II tests) for Figure 1_NO_results.

```
             Effect DFn DFd       F         p p<.05   ges
1          Genotype   5  24  36.700 1.75e-10     * 0.884
2         Condition   1  24 606.717 1.51e-18     * 0.962
3 Genotype:Condition   5  24  23.125 1.86e-08     * 0.828

  Genotype Effect      DFn   DFd     F         p p<.05   ges
1 Col-0    Condition     1    24  57.2 8.36e- 8 *       0.705
2 cue1-1   Condition     1    24  78.6 4.88e- 9 *       0.766
3 cue1-5   Condition     1    24  11.7 2.00e- 3 *       0.328
4 cue1-6   Condition     1    24 125.  5.47e-11 *       0.839
5 nox1     Condition     1    24 131.  3.23e-11 *       0.845
6 pOCA     Condition     1    24 319.  2.31e-15 *       0.93

  Condition Effect     DFn   DFd     F         p p<.05   ges
1 Control   Genotype     5    24 54.5  2.53e-12 *       0.919
2 NaCl      Genotype     5    24  5.28 2.00e- 3 *       0.524
```

ANOVA Table (type II tests) for Figure 5_NO_results.

```
             Effect DFn DFd       F         p p<.05   ges
1          Genotype   3  24 206.038 2.94e-17     * 0.963
2         Condition   1  24 336.363 1.27e-15     * 0.933
3 Genotype:Condition   3  24  33.987 8.37e-09     * 0.809

Genotype Effect      DFn   DFd     F         p p<.05   ges
1 Col-0    Condition     1    24  13.6 1.00e- 3 *       0.363
2 cue1-1   Condition     1    24  82.1 3.26e- 9 *       0.774
```

```
3 cue1-5    Condition      1     24 298.  4.85e-15 *        0.926
4 pOCA      Condition      1     24  44.3 6.97e- 7 *        0.649

 Condition Effect     DFn    DFd      F         p p<.05   ges
1 Glucose   Genotype     3     24 201.  4.02e-17 *        0.962
2 Sucrose   Genotype     3     24  39.5 1.94e- 9 *        0.832
```

ANOVA Table (type II tests) for Figure 6_MeristemCellNumber_results.

```
             Effect DFn DFd      F        p p<.05   ges
1          Genotype   3  70 40.279 3.08e-15     * 0.633
2         Condition   1  70 15.787 1.70e-04     * 0.184
3 Genotype:Condition   3  70  6.161 8.88e-04     * 0.209

Genotype Effect      DFn   DFd      F          p p<.05     ges
1 Col-0     Condition      1     70   0.022 0.881        ""       0.00032
2 cue1-1    Condition      1     70 26.3    0.00000252 "*"      0.273
3 cue1-5    Condition      1     70   6.94  0.01       "*"      0.09
4 pOCA      Condition      1     70   1.03  0.312       ""       0.015

 Condition Effect     DFn    DFd      F         p p<.05   ges
1 Glucose   Genotype     3     70   36.3 2.93e-14 *        0.609
2 Sucrose   Genotype     3     70   10.2 1.23e- 5 *        0.303
```

ANOVA Table (type II tests) for Figure 6_MeristemSize_results.

```
             Effect DFn DFd         F        p p<.05      ges
1          Genotype   3  70 27.330000 8.22e-12     * 5.39e-01
2         Condition   1  70  0.000408 9.84e-01       5.83e-06
3 Genotype:Condition   3  70  6.650000 5.13e-04     * 2.22e-01

Genotype Effect      DFn   DFd     F     p p<.05   ges
1 Col-0     Condition      1     70   2.48 0.12  ""       0.034
2 cue1-1    Condition      1     70 10.5  0.002 "*"      0.13
3 cue1-5    Condition      1     70   1.62 0.207 ""       0.023
4 pOCA      Condition      1     70   5.35 0.024 "*"      0.071

  Condition Effect     DFn    DFd     F         p p<.05   ges
1 Glucose   Genotype     3     70 29.0  2.59e-12 *        0.555
2 Sucrose   Genotype     3     70   4.93 4.00e- 3 *        0.174
```

Table S3. Protein content of 4-day-old and 7-day-old seedlings (μg/ml).

Protein Content of 4-day-old seedlings (μg/ml).

	Col-0	*cue1-5*	*cue1-6*	*nox1*	pOCA108	*cue1-1*
**Control**	5.27 ± 0.10	5.34 ± 0.24	5.48 ± 0.07	5.14 ± 0.09	5.37 ± 0.27	4.87 ± 0.13
**NaCl**	4.48 ± 0.15	4.94 ± 0.06	4.96 ± 0.04	5.30 ± 0.01	5.34 ± 0.13	4.92 ± 0.28

Protein Content of 7-day-old seedlings (μg/ml).

	Col-0	*cue1-5*	pOCA108	*cue1-1*
**Glucose**	3.37 ± 0.04	2.98 ± 0.17	3.18 ± 0.18	2.77 ± 0.09
**Sucrose**	3.33 ± 0.13	2.87 ± 0.25	3.43 ± 0.07	3.04 ± 0.13
